# Post-traumatic stress disorder after childbirth: A Tunisian study

**DOI:** 10.1192/j.eurpsy.2024.1379

**Published:** 2024-08-27

**Authors:** N. Sghaier, R. Ben Soussia, H. Bouchahda, I. Belhadj, H. Ben Garouia, H. Khiari

**Affiliations:** ^1^Psychiatry Departement; ^2^Gynecology and obstetrics Department, Taher Sfar Hospital, Mahdia; ^3^Psychiatry Departement, Ibn Jazzar Hospital, Kairouan, Tunisia

## Abstract

**Introduction:**

Childbirth is a special time for every woman, bringing pregnancy to an end and marking the birth of a new baby. This transitional event presents countless physical and psychological changes. Post-traumatic stress disorder (PTSD), the result of particularly intense stress, is often linked to the perception of childbirth as a traumatic event, requiring optimized follow-up and screening.

**Objectives:**

The aim of this current study is to estimate the prevalence of post-partum post-traumatic stress disorder in a sample group of Tunisian women and to determine factors associated with childbirth-related post-traumatic stress disorder.

**Methods:**

This is a longitudinal prospective descriptive study conducted among women hospitalized for childbirth in the obstetrics and gynecology department and those who consulted the prenatal outpatient clinic at Taher Sfar Mahdia Hospital. The duration of the study is 7 months, from March 15, 2020 to September 15, 2020. Data collection was based on a pre-established questionnaire determining the various socio-demographic and clinical characteristics. Psychometric assessment was carried out using the Posttraumatic Stress Disorder Checklist Scale (PCL-S).

**Results:**

We enrolled 120 women with a mean age of 28.2± 5.3 years. Few women had a psychiatric history of depression (1.2%) or anxiety (3%), and 29% had a pathological obstetric history. Nevertheless, 12.5% of patients were hospitalized during pregnancy. Eighty-seven patients expressed anticipatory fear of childbirth, and 102 women had good marital and social support. Almost half of deliveries (48.3%) were vaginal, and almost a third (27.5%) were emergency caesarean sections. Level 3 pain was reported in 73.3% of cases. Psychometric assessment revealed a prevalence of PTSD of 5.8%, with PTSD symptomatology in 18.4% of women. PTSD was statistically associated with low level of education (p=0.02), postpartum complications (p=0.05) and gender of newborn (p=0.01).

**Conclusions:**

Postpartum PTSD is a major public health problem affecting the healthy development of the newborn, the overall mental and physical recovery and well-being of the mother.Our findings suggest several intervention points for healthcare practitioners, including careful prenatal screening of past trauma history, social support, pain management and expectations about the birth, within a multidisciplinary approach.

**Disclosure of Interest:**

None Declared

